# Permanent draft genome of *Thermithiobaclillus tepidarius* DSM 3134^T^, a moderately thermophilic, obligately chemolithoautotrophic member of the *Acidithiobacillia*

**DOI:** 10.1186/s40793-016-0188-0

**Published:** 2016-09-26

**Authors:** Rich Boden, Lee P. Hutt, Marcel Huntemann, Alicia Clum, Manoj Pillay, Krishnaveni Palaniappan, Neha Varghese, Natalia Mikhailova, Dimitrios Stamatis, Tatiparthi Reddy, Chew Yee Ngan, Chris Daum, Nicole Shapiro, Victor Markowitz, Natalia Ivanova, Tanja Woyke, Nikos Kyrpides

**Affiliations:** 1 School of Biological Sciences, University of Plymouth, Drake Circus, Plymouth, PL4 8AA UK; 2 Sustainable Earth Institute, University of Plymouth, Drake Circus, Plymouth, PL4 8AA UK; 3 DOE Joint Genome Institute, Walnut Creek, CA 94598 USA

**Keywords:** *Thermithiobacillus tepidarius*, *Acidithiobacillia*, Sulfur oxidation, Chemolithoautotroph, Thiosulfate, DUF302

## Abstract

*Thermithiobacillus tepidarius* DSM 3134^T^ was originally isolated (1983) from the waters of a sulfidic spring entering the Roman Baths (Temple of Sulis-Minerva) at Bath, United Kingdom and is an obligate chemolithoautotroph growing at the expense of reduced sulfur species. This strain has a genome size of 2,958,498 bp. Here we report the genome sequence, annotation and characteristics. The genome comprises 2,902 protein coding and 66 RNA coding genes. Genes responsible for the transaldolase variant of the Calvin-Benson-Bassham cycle were identified along with a biosynthetic horseshoe *in lieu* of Krebs’ cycle *sensu stricto*. Terminal oxidases were identified, *viz.* cytochrome *c* oxidase (*cbb*_3_, EC 1.9.3.1) and ubiquinol oxidase (*bd*, EC 1.10.3.10). Metalloresistance genes involved in pathways of arsenic and cadmium resistance were found. Evidence of horizontal gene transfer accounting for 5.9 % of the protein-coding genes was found, including transfer from *Thiobacillus* spp. and *Methylococcus capsulatus* Bath, isolated from the same spring. A *sox* gene cluster was found, similar in structure to those from other *Acidithiobacillia* – by comparison with *Thiobacillus thioparus* and *Paracoccus denitrificans*, an additional gene between *soxA* and *soxB* was found, annotated as a DUF302-family protein of unknown function. As the Kelly-Friedrich pathway of thiosulfate oxidation (encoded by *sox*) is not used in *Thermithiobacillus* spp., the role of the operon (if any) in this species remains unknown. We speculate that DUF302 and *sox* genes may have a role in periplasmic trithionate oxidation.

## Introduction

*Thermithiobacillus tepidarius*DSM 3134^T^ [1, 2] is a moderately thermophilic sulfur-oxidising obligately chemolithoautotrophic member of the *Acidithiobacillia* originally published as ‘*Thiobacillus tepidarius*’ and assigned to the *Betaproteobacteria* – this has since been resolved by proteogenomic studies and the species moved firstly to a new genus – *Thermithiobacillus* – [3] in the *Gammaproteobacteria* and later to a separate Class, along the the genus *Acidithiobacillus* [4]. To date it is the only species of the genus with a validly published name and one of only two strains in cultivation [5]. The obligately aerobic chemolithoautotroph was demonstrated [1, 2] to use sulfur oxyanions as sole energy sources. It has a temperature optimum of 44 °C, reflecting its environment of isolation. Chemostat-based studies have demonstrated unusually high specific growth yields compared to other chemolithoautotrophs and biochemical studies have demonstrated the presence of a range of sulfur oxidation enzymes including rhodanese (EC 2.8.1.1), trithionate hydrolase (EC 3.12.1.1), thiosulfate dehydrogenase (EC 1.8.2.2), a tetrathionate-proton symport system [6] and at least 3 of each cytochromes *c* and *b* [7]. Proton translocations per mole of energy source were significantly higher than in other sulfur-oxidising autotrophs, potentially explaining the high yields. *T. tepidarius*DSM 3134^T^ was selected for genome sequencing as part of the Department of the Environment DOE-CSP 2012 initiative – as a type species of a genus.

## Organism information

### Classification and features

**Fig. 1:**
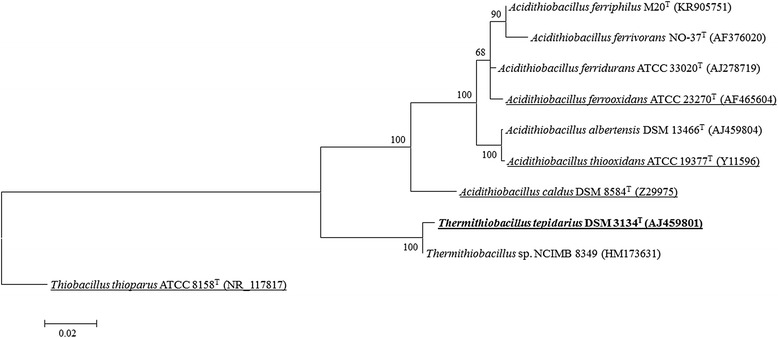
Maximum-likelyhood phylogenetic tree based on CLUSTALW alignment of 16S rRNA gene sequences of the *Acidithiobacillia*. Type strains of each species of *Acidithiobacillus* are used, along with that of *Thermithiobacillus* (emboldened)*. Thermithiobacillus* sp. NCIMB 8349 (the only other *Thermithiobacillus* sp. in culture) is given for the sake of completeness. Sequences pertaining to organisms for which a publically available genome sequence exists are underlined. Accession numbers for the GenBank database are in parentheses. Alignment and tree were constructed in MEGA 6 [30] using 1,509 positions and pairwise deletion. Tree was drawn using the Tamura-Nei model for maximum-likelyhood trees [31]. Values at nodes are based on 5,000 bootstrap replicates. Scale-bar indicates 2 substitutions per 100. *Thiobacillus thioparus* DSM 505^T^ is used as the outgroup

This strain was isolated from sulfidic groundwater flowing into a Roman bathhouse (Temple of Sulis-Minerva, now The Roman Baths, Bath, UK) – the only other strain of this genus held in a culture collection (*Thermithiobacillus* sp. NCIMB 8349) came from decomposing concrete in the Melbourne sewers in the 1940s [5]. The authors have detected at least 6 OTUs representing probably other *Thermithiobacillus* spp. in 16S rRNA gene libraries from the Roman Baths and have isolated a number of strains to date, indicating that *Thermithiobacillus* spp. are no more difficult to isolate than other sulfur-oxidising autotrophs and may thus simply be rare or confined to rare ecosystems. It forms white colonies of 2–5 mm diameter in 48 h that smell faintly of elementary sulfur if grown on thiosulfate-containing basal salts agar. In batch cultures, thiosulfate is oxidized stoichiometrically to tetrathionate early in the exponential phase, resulting in an increase in culture pH from pH 6.8 to pH 7.5–8.0 – a hallmark of the genus – before being fully oxidized to sulfate, with concomitant fall in culture pH, usually ending at pH 5.2. In continuous cultures, no intermediates accumulate in the medium. In the authors’ hands, trithionate has also been observed very early in the growth phase in batch culture, prior to tetrathionate production. Substrate-level phosphorylation appears not to participate in the energy conservation of this strain and all ATP is thus formed through oxidative phosphorylation [2]. The type – and only – strain was isolated from an enrichment culture comprising water obtained from the inflow of the Great Bath (Roman Baths, Bath, UK) in 1983 (Ann P. Wood, *personal communication*) added to a basal salts medium supplemented with thiosulfate and monomethylamine hydrochloride, before plating onto basalt salts agar containing 5 mM thiosulfate as sole energy source and incubated under air enriched with 5 % (*v/v*) carbon dioxide as sole carbon source. Key features of this organism are summarized in Table 1. A phylogenetic tree based on the 16S rRNA gene sequence, showing the position of the organism with regard to the *Acidithiobacillia*, rooted with *Thiobacillus thioparus*, is given in Fig. 1.

**Table 1:** Classification and general features of *Thermithiobacillus tepidarius* DSM 3134^T^ according to MIGS recommendations [8]

MIGS ID	Property	Term	Evidence code^a^
	Classification	Domain *Bacteria*	TAS [34]
		Phylum *Proteobacteria*	TAS [4, 35]
		Class *Acidithiobacillia*	TAS [4]
		Order *Acidithiobacillales*	TAS [4]
		Family *Thermithiobacillaceae*	TAS [4]
		Genus *Thermithiobacillus*	TAS [3]
		Species *Thermithiobacillus tepidarius*	TAS [1–5]
		(Type) strain: *DSM 3134* ^T^	TAS [1–5]
	Gram stain	Negative	TAS [1, 2]
	Cell shape	Rod	TAS [1, 2]
	Motility	Motile	TAS [1, 2]
	Sporulation	None	TAS [1, 2]
	Temperature range	20–52 °C	TAS [1, 2, 5]
	Optimum temperature	44 °C	TAS [1, 2]
	pH range; Optimum	5.2–8.0; 6.8	TAS [1, 2]
	Carbon source	Carbon dioxide	TAS [1, 2]
MIGS-6	Habitat	Thermal sulfidic springwater	TAS [1]
MIGS-6.3	Salinity	*N.D.*	NAS [1–5]
MIGS-22	Oxygen requirement	Aerobic	TAS [1, 2]
MIGS-15	Biotic relationship	Free-living	TAS [1, 2]
MIGS-14	Pathogenicity	Non-pathogen	NAS
MIGS-4	Geographic location	United Kingdom/England	TAS [1, 2]
MIGS-5	Sample collection	1983	NAS
MIGS-4.1	Latitude	51.381072	TAS [1, 2]
MIGS-4.2	Longitude	-2.359619	TAS [1, 2]
MIGS-4.4	Altitude	31 m	TAS [1, 2]

^a^Evidence codes - *IDA* Inferred from direct assay, *TAS* traceable author statement (i.e., a direct report exists in the literature), *NAS* non-traceable author statement (i.e., not directly observed for the living, isolated sample, but based on a generally accepted property for the species, or anecdotal evidence). These evidence codes are from the Gene Ontology project [28, 29]

**Fig. 2:**
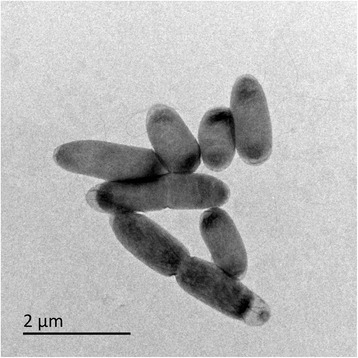
Transmission electron micrograph of *T. tepidarius* from a thiosulfate-limited chemostat (20 mM, 0.15 h^-1^). Cells were obtained from a chemostat-culture at steady-state by centrifugation and were washed and suspended in sterile 150 mM sodium chloride solution and applied to Formvar® and carbon coated copper grid before washing with further saline and staining in 50 mM uranyl acetate for 5 mins and washing again. Stained grids were visualized in a JEOL JEM-1400Plus transmission electron microscope, operating at 120 kV

Cells are 0.6 – 1.0 by 0.2 to 0.4 μm and stain Gram negative. They are rapidly motile by means of a single polar flagellum up to 4 μm in length, as shown in Fig. 2. Ubiquinone-8 is the dominant respiratory quinone and cells fix carbon dioxide *via* the Calvin-Benson-Bassham cycle at the expense of inorganic sulfur oxidation. Cells accumulate polyphosphate (‘volutin’) granules when grown in batch culture but are typically free from storage granules when grown in energy-source-limited chemostats. Anaerobic growth is not observed with tetrathionate as the electron donor and nitrate, nitrite, nitrous oxide, elementary sulfur, sulfate, tetrathionate or pyruvate as terminal electron acceptors, but cultures can reduce nitrate to nitrite. Experimental estimations of G + C content of genomic DNA are 66.6 ± 0.5 mol% by buoyant density [1] or 65.9 ± 0.8 mol% by acid denaturation [9] in our hands. Dry biomass is 47 % (*w/w*) C regardless of the energy source used. *T. tepidarius*DSM 3134^T^ does not grow on any organic carbon compound tested, including sugars (glucose, ribose, fructose, sucrose), intermediates of Krebs cycle (citrate, succinate, fumarate, malate, oxaloacetate), carboxylates (glycolate, formate, acetate, propionate, pyruvate), C_1_ compounds (monomethylamine, dimethylamine, trimethylamine, methanol, methane), structural amino acids (all 20), substituted thiophenes (thiophene-2-carboxylate, thiophene-3-carboxylate) or complex media (yeast extract, nutrient broth, brain-heart infusion, Columbia sheep blood agar, chocolate agar). Energy sources that support autotrophic growth are elementary sulfur, sulfide, trithionate, tetrathionate, hexathionate, heptathionate and thiosulfate. Fe(II), Mn(II), Cu(I), U(IV), pentathionate, dithionate, thiocyanate, sulfite, carbon disulfide, carbonyl sulfide, dimethylsulfide, dimethylsulfoxide, dimethylsulfone and formate do not support autotrophic growth as energy sources. The high growth yields and tetrathionate-accumulation in the early phases of growth make this strain a very interesting model organism for elucidation of sulfur oxidation pathways and their evolution.

## Genome sequencing information

### Genome project history

This organism was selected for sequencing on the basis of its role in sulfur cycling, physiological, biochemical, evolutionary and biogeochemical importance, and is part of the Genomic Encyclopedia of *Bacteria* and *Archaea*, 1,000 Microbial Genomes project at the U.S. Department of Energy, Joint Genome Institute (JGI). The genome project is deposited in the Genomes OnLine Database [10] and a high-quality permanent draft genome sequence in IMG [11]. Sequencing, finishing and annotation were performed by the JGI using state of the art sequencing technology [12]. A summary of the project information is shown in Table 2.

**Table 2:** Project information

MIGS ID	Property	Term
MIGS 31	Finishing quality	Improved High-Quality Draft
MIGS-28	Libraries used	Illumina Standard PE
MIGS 29	Sequencing platforms	Illumina HiSeq 2000/2500
MIGS 31.2	Fold coverage	116.4
MIGS 30	Assemblers	Allpaths/Velvet
MIGS 32	Gene calling method	NCBI Prokaryotic Genome Annotation Pipeline
	Locus Tag	G579DRAFT
	Genbank ID	AUIS01000000
	GenBank Date of Release	August 15, 2015
	GOLD ID	Ga0002306
	BIOPROJECT	PRJNA185671
MIGS 13	Source Material Identifier	DSM 3134^T^
	Project relevance	GEBA-KMG

### Growth conditions and genomic DNA preparation

*T. tepidarius*DSM 3134^T^ DNA was obtained from Dr Hans-Peter Klenk at the Deutsche Sammlung von Mikroorganismen und Zellkulturen Gmbh (DSMZ) having been grown on basal salts medium pH 6.9, supplemented with 10 mM tetrathionate as the sole energy source (DSM Medium 333). DNA was extracted using the JETFLEX Genomic DNA Purification Kit from Genomed (Löhne, Germany) into TE Buffer.

### Genome sequencing and assembly

The draft genome of *Thermithiobacillus tepidarius*DSM 3134^T^ was generated at the DOE Joint Genome Institute (JGI) using the Illumina technology [13]. An Illumina standard shotgun library was constructed and sequenced using the Illumina HiSeq 2000 platform which generated 13,370,056 reads totaling 2,005.5 Mbp. Library construction and sequencing were performed at the JGI - details are on their website [14]. All raw Illumina sequence data was passed through JGI’s DUK filtering program, which removes known Illumina sequencing and library preparation artifacts (Mingkun L, Copeland A, Han J, Unpublished). Filtered Illumina reads were assembled using Velvet (version 1.1.04) [15]; 1–3 Kbp simulated paired end reads were created from Velvet contigs using wgsim [16] and Illumina reads were assembled with simulated read pairs using Allpaths–LG (version r42328) [17]. Parameters for assembly steps were: Velvet (velveth: 63 –shortPaired and velvetg: −very clean yes –exportFiltered yes –min contig lgth 500 –scaffolding no –cov cutoff 10); wgsim (−e 0 –1 100 –2 100 –r 0 –R 0 –X 0); Allpaths–LG (PrepareAllpathsInputs: PHRED_64 = 1 PLOIDY = 1 FRAG_COVERAGE = 125 JUMP_COVERAGE = 25 LONG_JUMP_COV = 50, RunAllpathsLG: THREADS = 8 RUN = std_shredpairs TARGETS = standard VAPI_WARN_ONLY = True OVERWRITE = True). The final draft assembly contained 44 contigs in 43 scaffolds. The total size of the genome is 2.96 Mbp and the final assembly is based on 3,44.8 Mbp of Illumina data, which provides an average 116.4× coverage of the genome.

### Genome annotation

Genes were identified using Prodigal [18] as part of the DOE-JGI genome annotation pipeline [19]. The predicted CDSs were translated and used to search the National Center for Biotechnology Information non-redundant database, UniProt, TIGR-Fam, Pfam, KEGG, COG, and InterPro database. These data sources were combined to assert a product description for each predicted protein. tRNAScanSE was used to find tRNA genes and rRNA genes were found using searches against models of the ribosomal RNA genes built from SIVLA [20, 21]. Additional gene prediction analysis and functional annotation was performed within the IMG-ER platform [22, 23]. For each gene discussed in this publication, the annotation was manually checked against the GenBank® databased manual searches using the BLASTn and BLASTp algorithms - both of the gene from *T. tepidarius* and using the equivalent gene from members of the *Acidithiobacillia* or *Escherichia coli*.

## Genome properties

The genome of *T. tepidarius*DSM 3134^T^ is 2,958,498 bp-long with a 66.8 mol% G + C content (Table 3). Of the 2,968 predicted genes, 2,902 were protein-coding genes and 66 were RNA genes, including 2 rRNA operons. A total of 2,348 genes (79.1 %) were assigned a putative function. A total of 3.4 % were identified as pseudogenes – the remainder annotated as hypothetical proteins. The properties and the statistics of the genome are given in Table 3. The distribution of genes into COGs functional categories is presented in Table 4. The genome is one of the smaller genomes of those sequenced thus far from chemolithoautotrophic *Proteobacteria* (Table 5).

**Table 3:** Genome statistics

Attribute	Value	% of total
Genome size (bp)	2,958,498	100.00
DNA coding (bp)	2,664,218	90.05
DNA G + C (bp)	1,977,520	66.84
DNA scaffolds	43	
Total genes	2,968	100.00
Protein coding genes	2,902	97.78
RNA genes	66	2.22
Pseudo genes	102	3.43
Genes in internal clusters	116	3.99
Genes with function prediction	2,348	79.11
Genes assigned to COGs	2,048	69.00
Genes with Pfam domains	2,457	82.78
Genes with signal peptides	270	9.10
Genes with transmembrane helices	710	23.92
CRISPR repeats	1

**Table 4:** Number of genes associated with general COG functional categories

Code	Value	% age	Description
J	190	6.9	Translation, ribosomal structure and biogenesis
A	1	0.0	RNA processing and modification
K	80	2.9	Transcription
L	88	3.2	Replication, recombination and repair
B	2	0.1	Chromatin structure and dynamics
D	40	1.5	Cell cycle control, Cell division, chromosome partitioning
V	67	2.4	Defense mechanisms
T	131	4.8	Signal transduction mechanisms
M	200	7.3	Cell wall/membrane biogenesis
N	92	3.3	Cell motility
U	58	2.1	Intracellular trafficking and secretion
O	124	4.5	Posttranslational modification, protein turnover, chaperones
C	166	6.0	Energy production and conversion
G	102	3.7	Carbohydrate transport and metabolism
E	145	5.3	Amino acid transport and metabolism
F	65	2.4	Nucleotide transport and metabolism
H	128	4.7	Coenzyme transport and metabolism
I	71	2.6	Lipid transport and metabolism
P	159	5.8	Inorganic ion transport and metabolism
Q	31	1.1	Secondary metabolites biosynthesis, transport and catabolism
R	162	5.9	General function prediction only
S	138	5.0	Function unknown
-	920	33.5	Not in COGs

The total is based on the total number of protein coding genes in the genome

**Table 5:** Genome properties of obligately chemolithoautotrophic members of the *Proteobacteria*

	Genome size (bp)	Protein encoding genes	CRISPR repeats	RNA genes
*Thermithiobacillus tepidarius* DSM 3134^T^	2,958,498	2,902	1	66
*Acidithiobacillus thiooxidans* ATCC 19377^T^	3,019,868	3,080	0	47
*Acidithiobacillus ferrooxidans* ATCC 23270^T^	2,982,327	3,147	1	87
*Acidithiobacillus caldus* ATCC 51756^T^	2,946,159	2,821	3	53
*Thiobacillus thioparus* DSM 505^T^	3,201,518	3,197	2	62
*Thiobacillus denitrificans* DSM 12475^T^	3,609,948	3,545	1	106
*Halothiobacillus neapolitanus* ATCC 23641^T^	2,582,886	2,413	1	52

## Insights from the genome sequence

As an obligate autotroph, it would be anticipated that genes encoding a complete Calvin-Benson-Bassham cycle and, *in lieu* of Krebs’ cycle, a biosynthetic horseshoe [24] would be present. A complete CBB cycle is present, and owing to the presence of a transaldolase (EC 2.2.1.2) and absence of a sedoheptulose-1,7-bisphosphatase (EC 3.1.3.37) gene, we can conclude that it is a transaldolase-variant CBB cycle [25]. Of Krebs’ cycle genes, citrate synthase (EC 2.3.3.16), aconitase (EC 4.2.1.3), isocitrate dehydrogenase (NADP^+^, EC 1.1.1.42), succinyl coenzyme A synthase (ADP-forming, EC 2.6.1.5) and malate dehydrogenase (oxaloacetate decarboxylating, NADP^+^, EC 1.1.1.40) were present. No fumarase or succinate dehydrogenase genes could be identified. The E1 subunit of α-ketoglutarate dehydrogenase was missing and the closest BLASTp match to the E2 subunit is annotated as a pyruvate dehydrogenase. These lesions are consistent with other obligate autotrophs and confirm the presence of a biosynthetic horseshoe in *T. tepidarius* [24].

In terms of respiration, 2 cytochrome *c* oxidases (*cbb*_3_ EC 1.9.3.1) and 2 ubiquinol oxidases (*bd*, EC 1.10.3.10) could be identified, which is consistent with previous physiological studies [7]. Three cytochromes *b*_561_ and three cytochromes *c*_553_ were identified, along with other cytochromes *c*, again constant with previous studies [7].

## Extended insights

**Fig. 3:**
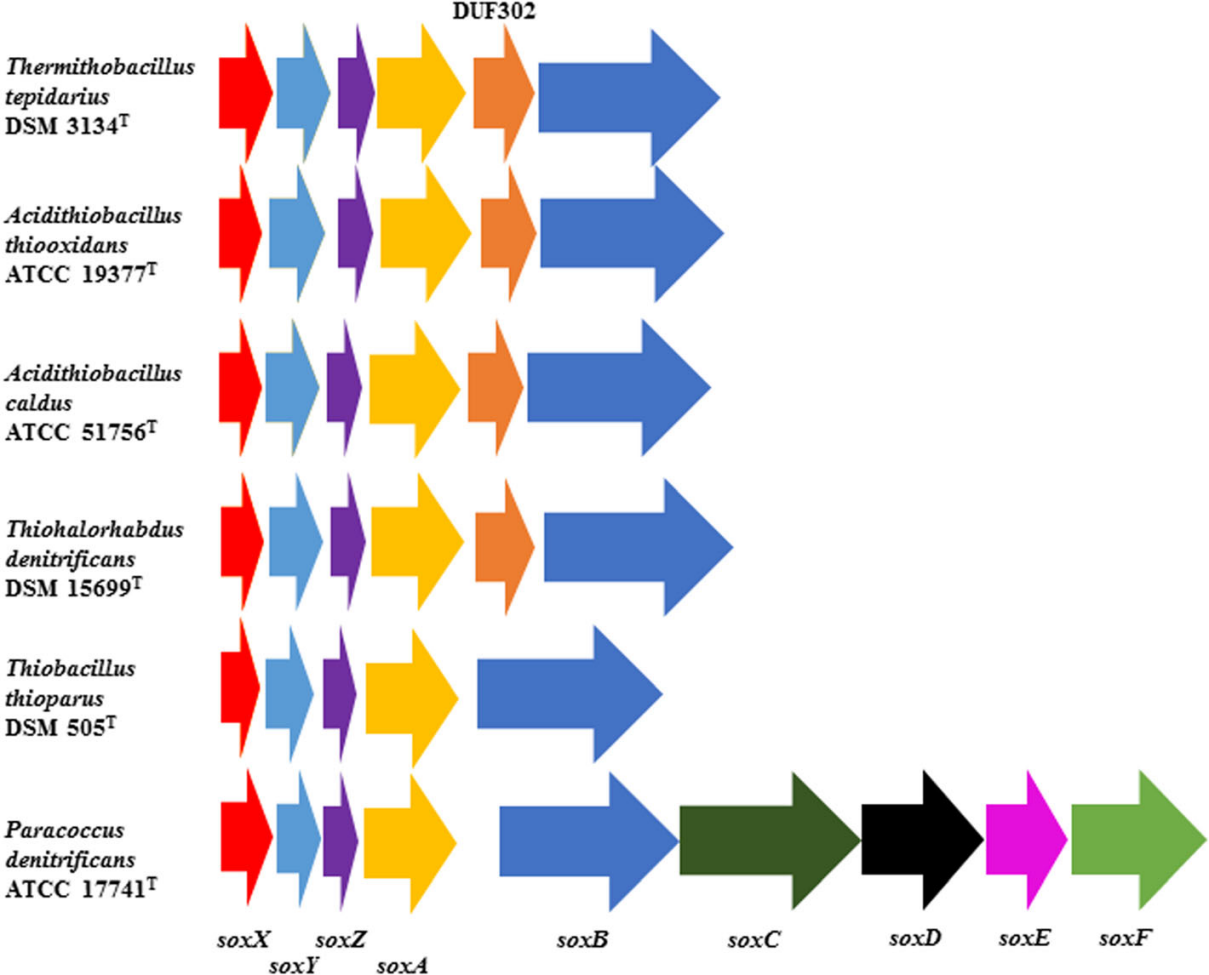
Structure of the *sox* cluster in *T. tepidarius* and other chemolithoautotrophic *Proteobacteria*. A reference *sox* operon encoding the Kelly-Friedrich pathway of thiosulfate oxidation from *Paracoccus denitrificans* ATCC 17741^T^ (*Alphaproteobacteria*) is given, showing *soxXYZABCDEF* genes and intergenic spacers, against gene clusters from *T. tepidarius* DSM 3134^T^, *A. thiooxidans* ATCC 19377^T^ and *A. caldus* ATCC 51756^T^ (the only sulfur-oxidising *Acidithiobacillus* spp.) of the *Acidithiobacillia*; *Thiohalorhabdus denitrificans* DSM 15699^T^ (*Gammaproteobacteria*) and *Thiobacillus thioparus* DSM 505^T^ (*Betaproteobacteria*). The DUF302-family hypothetical protein gene is indicated where present. It is worth noting that *P. denitrificans* and *T. thioparus* do not grow on trithionate and that thiosulfate oxidation in *Thermithiobacillus* and *Thiobacillus* has been unequivocally shown not to proceed *via* the periplasmic Kelly-Friedrich oxidation pathway and instead occurs *via* tetrathionate as an intermediate, which is then oxidized to sulfate in the cytoplasm (the Kelly-Trudinger pathway [32, 33]). The highly conserved *soxXYZAB* cluster occurs in all of the genomes examined and the DUF302 gene appears highly conserved in the *Acidithiobacillia*. The function is as-yet unknown, as is that of the *sox* genes in these Kelly-Trudinger pathway organisms. Analysis of conserved domains indicates that DUF302 may form a homodimer

Two pairs of genes encoding ribulose-1,6-bisphosphate carboxylase (RuBisCO) could be identified, each comprising a large and small subunit gene. One pair is found close to *cbbO* and *cbbQ* genes, with no other *cbb* genes closeby – this is consistent with *Acidithiobacillus* spp. and other obligate chemolithoautotrophs and indicates a Form IAq RuBisCO. The other pair is found close to *cbb* genes and in that sense is perhaps more similar to Form II RuBisCO [26]. Metalloresistance genes including those for arsenite efflux and arsenate reductase (*arsB* and *arsC*, respectively) were identified along with those implicated in tellurite, cadmium, cobalt, zinc, copper and silver resistance. Sulfur-oxidation genes are obviously of paramount interest in an obligate chemolithoautotroph, however, a number of proposed enzymes of sulfur metabolism have no genes identified thus far. It is known that the *Acidithiobacillia* [1, 2, 4–6] do not use the Kelly-Friedrich or “Sox” pathway of thiosulfate oxidation, and instead oxidise thiosulfate to tetrathionate *via* a poorly understood dehydrogenase – more than one form of which may exist. Some Kelly-Friedrich pathway genes are present in the genome and these are given in Fig. 3, showing comparison with those from other organisms that do not use the Kelly-Friedrich pathway *versus* one (*Paracoccus denitrificans*) that does. It can be seen from Fig. 3 that the non-Kelly-Friedrich organisms lack the *soxC* and *soxD* genes that are involved in a 6-electron capture during thiosulfate oxidation and all contain a gene encoding DUF302-family protein of unknown function 191 amino acids in length (G579DRAFT_01426 in *T. tepidarius*). Assuming these proteins are found in the periplasm of *T. tepidarius* as they are in *Paracoccus* spp., they could play a role in trithionate or higher polythionate oxidation (tetrathionate being oxidized solely in the cytoplasm [6]. The DUF302 protein of *T. tepidarius* would have a mass of 20.6 kDa based on the amino acyl sequence but contains a potential dimerization domain, so could be 41.2 kDa. It is worth noting that the periplasmic trithionate hydrolase (EC 3.12.1.1, gene unknown) of *Acidiphilium acidophilum* was 35 kDa [27].

One hundred seventy eight genes (5.9 % of genome) were flagged as potentially horizontally transferred from the species *Thiobacillus thioparus*, *Thiobacillus denitrificans* and *Sulfuricella denitrificans* in the *Hydrogenophilaceae*. This is particularly interesting since *Thiobacillus aquaesulis*DSM 4255^T^ (= ATCC 43788^T^, no genome available) is closely related to these 3 species and was isolated originally from the Roman Baths and thus inhabits the exact same location [28]. A further 55 genes (1.9 %) were potentially transferred from *Methylococcus**capsulate,* a strain of which (Bath = NCIMB 11132) was also isolated from the Roman Baths [25]. There is no clear pattern in the proteins encoded by the genes marked as potentially transferred.

## Conclusions

The genome of *T. tepidarius*DSM 3134^T^ is the first for this genus and one of very few available for the Class *Acidithiobacillia*. The genome gives evidence and insight into the carbon dioxide fixation pathway, biosynthesis and sulfur oxidation as well as metal resistance and potential gene transfer from other species also isolated from the Roman Baths from which this organism was obtained. These data confirm that a transaldolase variant of the Calvin-Benson-Bassham cycle is used for carbon dioxide fixation. Sulfur oxidation genes of the *sox* operon are present but *soxC* and *soxD* are missing, though a DUF302-family protein was present – and also found across obligate chemolithoautotrophs in the *Proteobacteria* that use the Kelly-Trudinger (aka S_4_I pathway) of sulfur oxidation, rather than the Kelly-Friedrich (aka Sox) pathway. This genome sequence has already been utilized to propose the Class *Acidithiobacillia* [4] for *Thermithiobacillus* and *Acidithiobacillus* and to determine their evolutionary relationship with the *Gammaproteobacteria*. Thus far, the type species of each genus of the *Acidithiobacillia* is now sequenced, along with several other *Acidithiobacillus* spp. and other obligate chemolithoautotrophic *Bacteria* such as *Thiobacillus* spp. and *Halothiobacillus* spp. (Table 5), of these, *T. tepidarius*DSM 3134^T^ has one of the smaller genomes, presumably because it lacks the salt-tolerance systems of *Halothiobacillus* spp. or the iron-oxidation or acid-tolerance of *Acidithiobacillus* spp. This genome sequence will enable further evolutionary studies into the nature of the *Acidithiobacillia* and chemolithoautotrophs in general, along with ecological studies including organism-organism interactions in the environment owing to the evidence for horizontal gene transfer evident in this genome.

## Abbreviations

KMG: 1,000 microbial genomes
S_4_I: Tetrathionate intermediate pathway (aka Kelly-Trudinger pathway)
Sox: Sulfur oxidation pathway (aka Kelly-Friedrich pathway)
