# Erratum to: Permanent draft genome of *Thermithiobacillus tepidarius* DSM 3134^T^, a moderately thermophilic, obligately chemolithoautotrophic member of the *Acidithiobacillia*

**DOI:** 10.1186/s40793-016-0197-z

**Published:** 2016-10-11

**Authors:** Rich Boden, Lee P. Hutt, Marcel Huntemann, Alicia Clum, Manoj Pillay, Krishnaveni Palaniappan, Neha Varghese, Natalia Mikhailova, Dimitrios Stamatis, Tatiparthi Reddy, Chew Yee Ngan, Chris Daum, Nicole Shapiro, Victor Markowitz, Natalia Ivanova, Tanja Woyke, Nikos Kyrpides

**Affiliations:** 1 School of Biological Sciences, University of Plymouth, Drake Circus, Plymouth, PL4 8AA UK; 2 Sustainable Earth Institute, University of Plymouth, Drake Circus, Plymouth, PL4 8AA UK; 3 DOE Joint Genome Institute, Walnut Creek, CA 94598 USA

## Erratum

*n.b. The errors and associated corrections described in this document concerning the original manuscript were accountable to the production department handling this manuscript, and thus are no fault of the authors of this paper. Additionally, the online manuscript has now been updated with these corrections accordingly.*

In the original publication of this article [1], the title was displayed incorrectly as “Permanent draft genome of *Thermithiobaclillus tepidarius* DSM 3134^T^, a moderately thermophilic, obligately chemolithoautotrophic member of the *Acidithiobacillia”.* The genus name “*Thermithiobacillus”* was misspelt as “*Thermithiobaclillus”.* This has now been corrected in the original article.
